# Melatonin alleviates depression-like behaviors and cognitive dysfunction in mice by regulating the circadian rhythm of AQP4 polarization

**DOI:** 10.1038/s41398-023-02614-z

**Published:** 2023-10-06

**Authors:** Di Yao, Rong Li, Jiahuan Hao, Hongqing Huang, Xubiao Wang, Lusen Ran, Yuanyuan Fang, Yuqin He, Wei Wang, Xinghua Liu, Minghuan Wang

**Affiliations:** 1 Department of Neurology, Tongji Hospital, Tongji Medical College, Huazhong University of Science and Technology, Wuhan, 430030 China; 2 Department of Pediatrics, Tongji Hospital, Tongji Medical College, Huazhong University of Science and Technology, Wuhan, 430030 China; 3 Hubei Key Laboratory of Neural Injury and Functional Reconstruction, Huazhong University of Science and Technology, Wuhan, 430030 China; 4 Key Laboratory of Neurological Diseases of the Chinese Ministry of Education, School of Basic Medicine, Tongji Medical College, Huazhong University of Science and Technology, Wuhan, 430030 China; 5 Trauma Centre/ Department of Emergency and Trauma Surgery, Tongji Hospital, Tongji Medical College, Huazhong University of Science and Technology, Wuhan, 430030 China

**Keywords:** Depression, Molecular neuroscience

## Abstract

Depression is a common chronic psychiatric illness, which is resistant to medical treatments. While melatonin may alleviate certain depression symptoms, evidence for its efficacy against core symptoms is lacking. Here, we tested a mechanism whereby melatonin rescues the behavioral outcomes of the chronic unpredictable mild stress (CUMS) mouse model of depression. CUMS mice showed depressive behaviors to tail suspension, open field behavior, and sucrose preference test, and cognitive dysfunction in the Morris water maze. Impairments in these measures were relieved by melatonin treatment. Moreover, CUMS mice had impaired glymphatic function across the sleep-wake cycle due to the astrocytic loss and disturbance of circadian regulation of the polarized expression of aquaporin-4 (AQP4) water channels in perivascular astrocytes. EEG results in CUMS mice showed a reduced total sleep time and non-rapid eye movement (NREM) sleep, due to sleep fragmentation in the light phase. CUMS mice lost the normal rhythmic expressions of circadian proteins Per2, Cry2, Bmal1, Clock, and Per1. However, the melatonin treatment restored glymphatic system function and the polarization of AQP4, while improving sleep structure, and rectifying the abnormal expression of Per2, Bmal1, Clock, and Per1 in CUMS mice. Interestingly, Per2 expression correlated negatively with the polarization of AQP4. Further studies demonstrated that Per2 directed the location of AQP4 expression via interactions with the α-dystrobrevin (Dtna) subunit of AQP4 in primary cultured astrocytes. In conclusion, we report a new mechanism whereby melatonin improves depression outcomes by regulating the expression of the circadian protein Per2, maintaining the circadian rhythm of astrocytic AQP4 polarization, and restoring glymphatic function.

## Introduction

Major depressive disorder (MDD) is a common, chronic, and potentially life-threatening illness with an increasing prevalence that affects the quality of life of millions of people worldwide [1–3]. The overall lifetime risk of MDD is 15–18%, and the 12-month prevalence of MDD is approximately 6% in the world [1]. Despite decades of research, highly effective medical treatments are lacking, perhaps due to insufficient understanding of the complex and heterogeneous pathogenesis of MDD [1, 4–6].

The core symptom of MDD is the disturbance of sleep structure, including delayed sleep onset, non-restful sleep, and early-morning waking, along with associated clinical symptoms such as daytime fatigue, and diminished normal morning peaks in subjective energy, mood, and alertness [7]. Maintenance of the central daily sleep-wake cycle, known as circadian rhythm, is disrupted in major depression, atypical depression, and seasonal affective disorder [7]. During sleep, toxic brain metabolites are cleared from the brain via the glymphatic system [8], and memory consolidation occurs [9]. As such, recovering the normal sleep architecture is a critical aspect of treating depression, and avoiding long-term sequelae of persistent sleep disturbances.

In recent years, the role of the glymphatic system in maintaining central nervous system homeostasis has attracted increasing attention. The glymphatic system is a bulk clearance pathway through the perivascular space, which is formed by astrocytic endfeet wrapped around penetrating arteries and arterioles [10]. The polarized expression of the water channel aquaporin-4 (AQP4), exports interstitial fluid to cerebrospinal fluid (CSF), which ultimately drains into the peripheral vasculature and lymphatic vessels of cranial nerves [11]. Altering AQP4 vascular polarization impairs the function of the glymphatic system [10]. Interestingly, glymphatic function exhibits a circadian rhythm in mice, with a peak around mid-day when mice are most likely to be asleep [12]. This circadian rhythm of glymphatic function is obtained in part via a rhythmic alteration of astrocytic AQP4 polarization, which is supported by rhythmic gene expression of key components in the dystrophin-associated complex (DAC) [12]. In animal models of depression, such as the chronic unavoidable mild stress (CUMS) model, the function of the glymphatic system is disrupted, which is associated with behavioral signs of depression and cognitive dysfunction [13].

The mainstay medical treatment of MDD consists of antidepressant medications, including serotonin-selective reuptake inhibitors. However, available treatments are only adequate for about one-third of MDD patients, and have their own adverse effects on circadian cycles and sleep structure [14]. The regulation of the circadian system by the pineal hormone melatonin has attracted renewed interest in exploring its potential clinical benefits [7]. Therefore, in the present study, we aimed to investigate the effects of melatonin in modulating circadian rhythms and the glymphatic system in a CUMS mouse model of depression.

## Results

### Melatonin improves the behavioral outcomes after CUMS

**Fig. 1:**
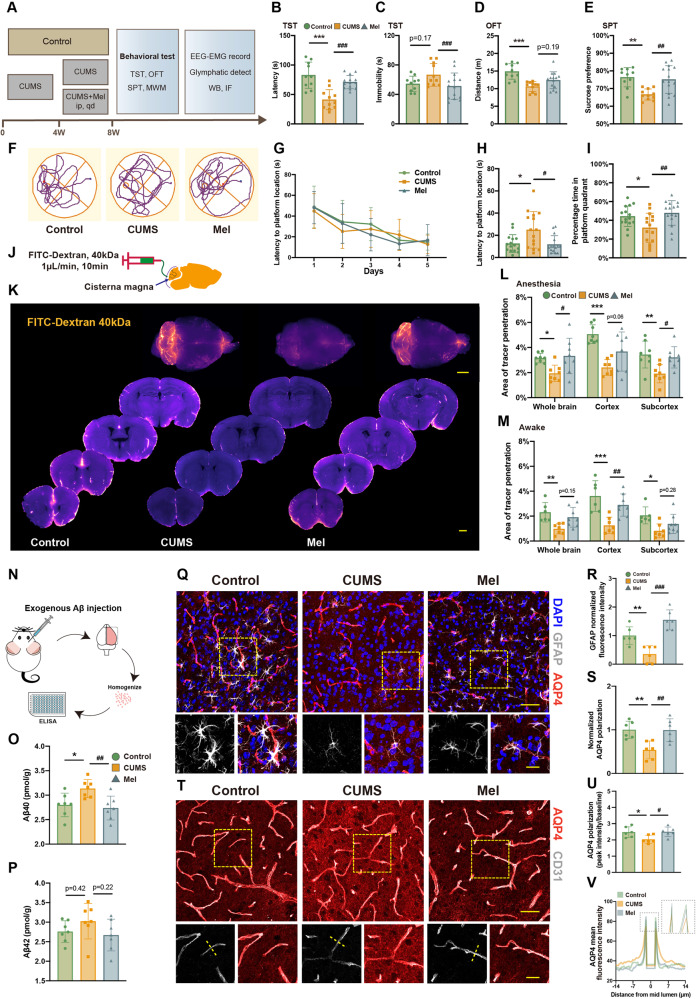
Melatonin treatment ameliorates depression-like behavior, cognitive deficits, glymphatic dysfunction, and AQP4 dislocation in CUMS mice. **A** Schematic diagram of the behavioral experimental procedure. **B**, **C** The latency to the first immobility and the duration of total immobility in the tail suspension test (TST). **D** The total travel distance during the open field test (OFT). **E** The percentage of sucrose consumption during the sucrose preference test (SPT). Control, *n* = 10; CUMS, *n* = 11; Mel, *n* = 14. **F** Tracing of locomotion for representative animals during the arena test. **G** The latency to finding the hidden platform in the Morris Water Maze test during the 5-days training. **H**, **I** The latency to finding the platform area and the percentage time spent in the platform quadrant in the probe test. Each group, *n* = 15. **J**–**V** Measurement of glymphatic function and AQP4 polarization. **J** Diagram of CSF tracer injection via the cisterna magna. **K** Representative images of CSF tracers in the whole brain and coronal slices. Scale bar, 2 mm. **L**, **M** Analysis of fluorescent area in the brains for the anesthesia and awake states. Anesthesia, each group *n* = 8. Awake, Control, *n* = 6; CUMS, *n* = 7; Mel, *n* = 8. **N** Diagram of stereotactic injection of exogenous mixture of human Aβ40 and Aβ42. **O**, **P** The brain concentration of exogenous human Aβ40 and Aβ42 measured by ELISA. Each group *n* = 7. **Q** Representative images of AQP4, DAPI, and GFAP immunostaining. **T** Representative images of AQP4 and CD31 immunostaining. Scale bars, 50 μm and 25 μm. **R** Quantitation of GFAP fluorescent intensity. **S** Quantitation of AQP4 polarization to **Q**. AQP4 polarization = donut-shaped area AQP4 / global AQP4. **U** Quantitation of AQP4 polarization to **T**. AQP4 polarization = vessel AQP4 (peak of the vertical line) / global AQP4. Collect 3–5 vessels from each of 6 mice (**R**, **S** and **U**). **V** Model of AQP4 fluorescence intensity plot along the yellow dotted line in (**T**). Signal within the vessels is the baseline intensity of AQP4. * #<0.05, ** ##<0.01, *** ###<0.001.

Two-month-old C57/BL male mice were subjected to CUMS paradigm for 8 weeks, as previously described [13]. Mice in the melatonin treatment group received melatonin treatment commencing in the fifth week of the CUMS procedure (Fig. 1A). We monitored the anal temperature of the mice for 24 h after each exposure to the CUMS procedure to confirm the absence of hypothermia in the CUMS mouse models (Fig. S1A–F). The tail suspension test (TST), open field test (OFT), and sucrose preference test (SPT) were performed to evaluate learned helplessness, anxiety, and anhedonia during the development of the CUMS model. In the TST, the CUMS group presented a shorter immobility latency compared to the control group, which was indicative of behavioral despair. Melatonin treatment significantly shortened the duration of immobility and prolonged the latency to immobility as compared to the untreated CUMS group. In the OFT, the CUMS group exhibited a shorter distance of movement compared to the control group. Melatonin administration enhanced the total distance covered by CUMS mice to an amount matching the control group but did not show a difference to the CUMS group. In the SPT, the CUMS group exhibited a lower preference for sucrose solution compared to the control group, which was normalized in the CUMS group with melatonin treatment (Fig. 1B–E). These data indicate that the CUMS paradigm established a behavioral model of depression, and that prolonged melatonin administration improved scores for depressive behaviors.

Next, we measured spatial memory performance with the Morris water maze (MWM). The three groups of mice showed similar learning curves in the five-day training period. The probe test, in which the platform was removed on the test day at one day after training, showed that the latency to find the location was longer in the CUMS group as compared to controls, whereas the percentage of time spent in the target quadrant was less, indicating an impairment of spatial learning in the CUMS animals. However, corresponding MWM test scores in the melatonin treated CUMS group were within the range for control animals (Fig. 1F–I). The results of the MWM test suggest that melatonin treatment rescued the impairment in spatial learning in CUMS mice.

### Melatonin improves the functions of the glymphatic system in CUMS mice

Next, we investigated the effects of melatonin treatment on glymphatic function. In the anesthesia state (sleep), the CUMS group showed a remarkable reduction in glymphatic influx, which was substantially rescued by administration of melatonin, notably in the whole brain and subcortex. In the awake state, the glymphatic influx was substantially impaired in the CUMS group. Melatonin treatment substantially rescued glymphatic influx in the cerebral cortex, but not in the whole brain and subcortex (Fig. 1J–M). Thus, administration of melatonin ameliorates the impairments of glymphatic influx in CUMS mice, especially so during the anesthesia state.

Next, we tested the efflux function of the glymphatic system by measuring the residual β-amyloid (Aβ) in the brain at one hour after injecting a mixture of human Aβ40 or Aβ42 into the prefrontal cortex. The ELISA results showed a deficit in clearing Aβ40 from the brain in the CUMS group, whereas Aβ42 clearance was unimpaired. Melatonin treatment rescued the capacity to clear Aβ40 in CUMS mice (Fig. 1N–P).

Perivascular astrocytes at the blood-brain barrier are the cellular foundation of the glymphatic system [10]. We found a reduced astrocytic density and shortened processes in the CUMS group (Figs. 1R, S3A–D), which was restored after melatonin treatment. Of note, immunostaining for the astrocytic specific marker glial fibrillary acidic protein (GFAP) revealed that the administration of melatonin restored the astrocyte numbers in the CUMS brains, thus sustaining a key component of the glymphatic system. AQP4 covers the astrocytic endfeet, with a polarized expression that facilitates the flow of fluid from the perivascular space to the brain parenchyma and then along perivascular channels. We analyzed the polarization of AQP4 by evaluating the AQP4 ratio and its association with surrounding capillaries. The AQP4 ratio, which is an index of polarization, was decreased in CUMS mice but was normalized after melatonin treatment (Fig. 1Q–S). To evaluate the association of AQP4 expression with vessels, we analyzed the AQP4 immunostaining density (green) around arterioles labeled for the vascular marker CD31 (vessel, red). The CUMS group showed significantly reduced polarization of AQP4 expression, which was restored by melatonin treatment (Fig. 1T–V). In summary, melatonin rescued the decreased AQP4 polarization seen in the CUMS mice.

### Melatonin improves the sleep quality of the CUMS group

**Fig. 2:**
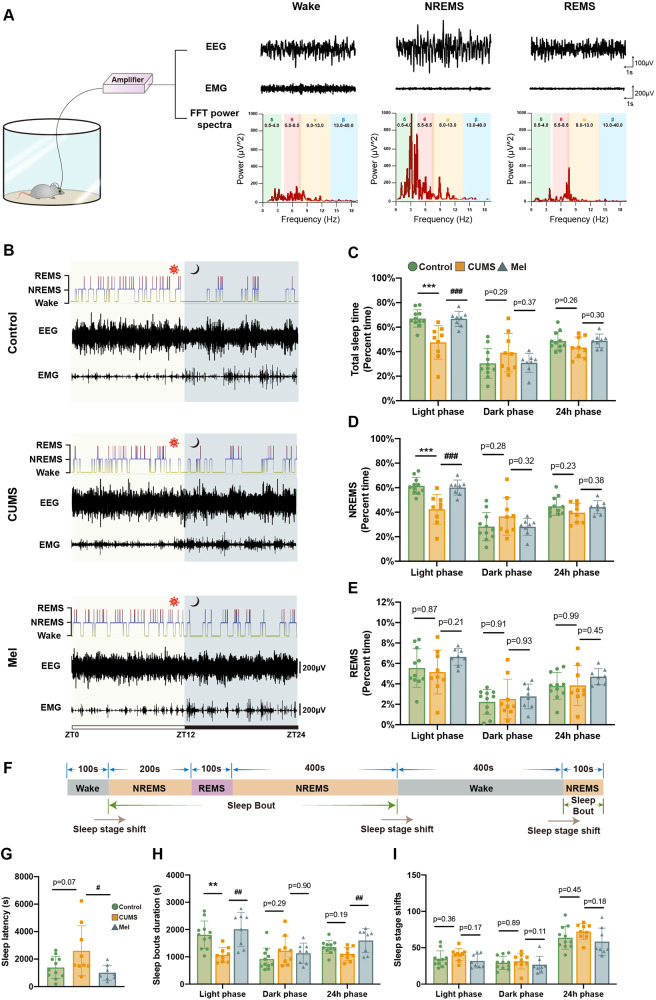
Melatonin treatment ameliorates the disturbance of sleep architecture in CUMS mice, especially during the light phase. **A** Illustration of EEG/EMG recordings and typical EEG, EMG, and FFT (fast Fourier transform) spectra in awake, NREMS, and REMS sleep phases. **B** Representative hypnograms, EEG, and EMG recording over 24 h. **C**–**E** Percentage of time for sleep, NREMS, and REMS in the light phase (ZT0-ZT12), dark phase (ZT12-ZT24), and entire 24 h phase (ZT0-ZT24). **F** Representative 1300 s segment of a hypnogram, illustrating a sleep bout and sleep stage shift. **G** Sleep latency (time from ZT0 to the appearance of the first sleep bout) in each group. **H**, **I** Quantitation of sleep bout duration and sleep stage shifts in the light phase, dark phase, and the entire 24-hour epoque. Control, *n* = 11; CUMS, *n* = 9; Mel, *n* = 7. * # <0.05, ** ##<0.01, *** ###<0.001.

Melatonin closely regulates the circadian rhythm and can enhance sleep quality [15, 16]. The results above indicate that melatonin enhanced glymphatic function, especially during sleep. Therefore, we analyzed effects of melatonin on sleep architecture in CUMS mice by recording the EMG-EEG (Fig. 2A).

In the light phase (rest state), the total sleep time and the duration of non-rapid eye movement (NREM) phase were reduced in the CUMS group but were restored to normal in the melatonin treatment group. In the dark phase (activity state), the total sleep time, NREM duration, and rapid eye movement (REM) sleep did not differ in the three groups of mice, nor did the total sleep time in 24 h (Fig. 2B–E). These results reveal that melatonin treatment rescued the alteration of sleep rhythm in the CUMS group, especially in the light phase.

We used the following factors to measure sleep quality of the mice: the time lag to the onset of the first sleep state (sleep latency), the average duration of each sleep period (sleep bout duration), and the number of sleep-awake transitions (sleep stage shift) (Fig. 2F). The latency to sleep tended to increase in the CUMS group compared to controls and was significantly shortened by melatonin treatment (Fig. 2G). Analysis of the 24 h- and the light phase recordings showed that sleep bout duration was decreased in the CUMS group, and was enhanced by the administration of melatonin (Fig. 2H). The sleep bout duration in the dark phase did not differ between the three groups, nor did we observe any group differences in the number of sleep-awake conversions (Fig. 2I). These results suggest that melatonin improved the sleep quality in CUMS mice by enhancing sleep bout duration, but without altering the number of sleep bouts.

**Fig. 3:**
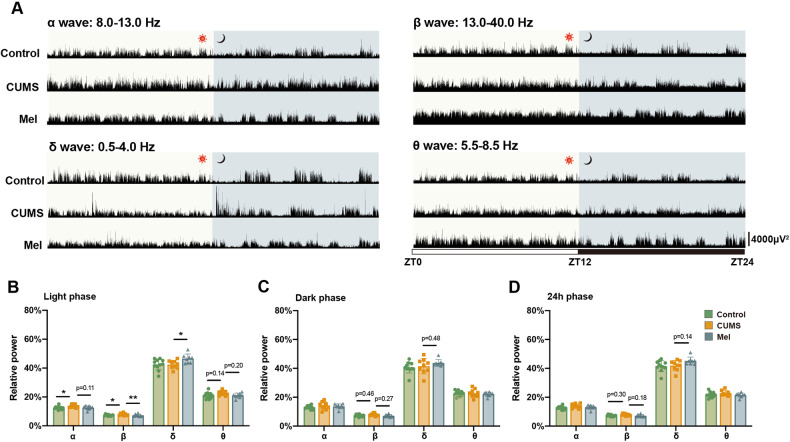
Melatonin treatment ameliorates the disturbance of sleep architecture in CUMS mice, especially during the light phase. **A** Representative EEG power spectra for α (8.0–13.0 Hz), β (13.0–40.0 Hz), δ (0.5–4.0 Hz), and θ (5.5–8.5 Hz) bands during 24 h. **B**–**D** Quantitation of α, β, δ, and θ wave relative power in light phase, dark phase, and an entire 24 h epoque. Control, *n* = 11; CUMS, *n* = 9; Mel, *n* = 7. * # <0.05, ** ## <0.01.

Since glymphatic system is closely related to the EEG power [17], we then analyzed the relative powers of EEG waves. In the light phase EEG recordings, the relative percentages of alpha and beta power were higher in the CUMS group, whereas the administration of melatonin restored the percentage of beta power to normal levels. The relative percentage of delta power was higher in the melatonin group than that in the CUMS and control groups, but did not differ between the CUMS and control groups (Fig. 3A–D).

### Melatonin improves the sleep quality of the CUMS mice via regulating Per2 expression

**Fig. 4:**
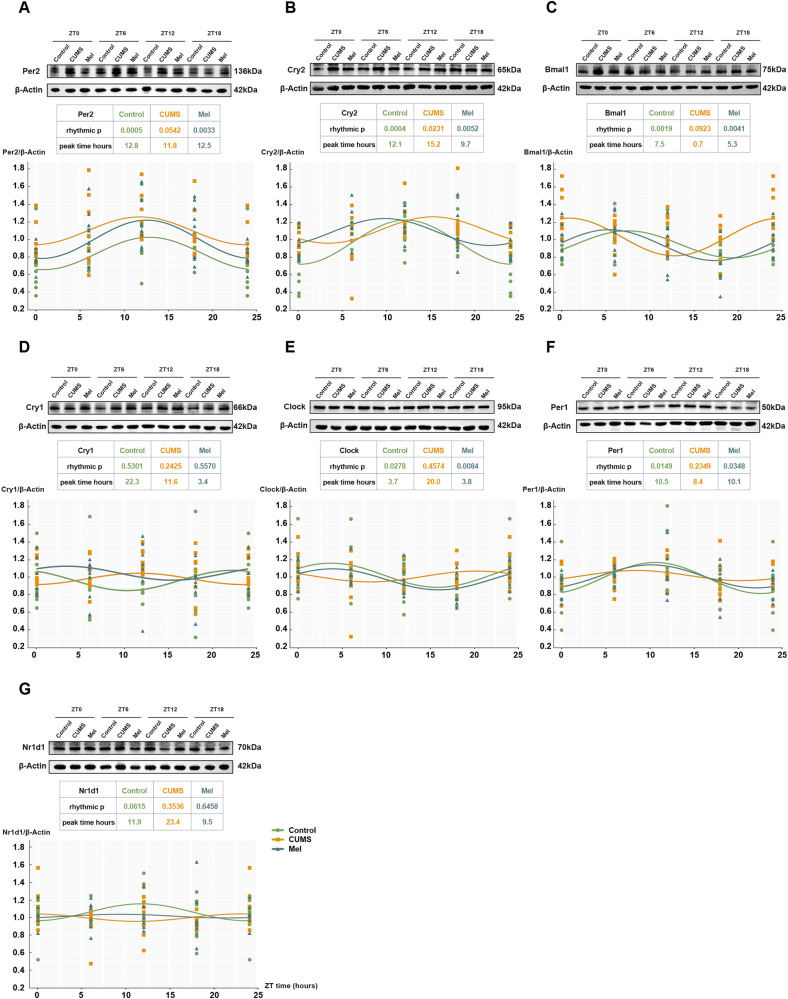
Melatonin treatment rescues the disturbances of expression of circadian proteins in CUMS mice. **A-G.** Representative western blots and corresponding quantifications of circadian proteins, including Per2, Cry2, Bmal1, Cry1, Clock, Per1, and Nr1d1 at ZT0, ZT6, ZT12, ZT18, respectively. Each group, *n* = 8.

Due to the pivotal role of circadian proteins in the regulation of the sleep-wake circle, we next analyzed the circadian rhythm of well-known circadian proteins (Clock, Bmal1, Cry1, Cry2, Per1, Per2 and Nr1d1) every 6 h, namely at Zeitgeber time (ZT)0, ZT6, ZT12, and ZT18 after the start of the light phase (ZT0) (Fig. 4A–G). Antibodies were validated in Fig. S4–S6. Both the control and melatonin groups exhibited distinct circadian rhythms for the expressions of Per2, Cry2, Bmal1, Clock, and Per1, with a rhythmic *p*-value of <0.05. Notably, Per2 and Cry2 demonstrated the lowest rhythmic *p*-values (0.0005 and 0.0004, respectively). However, CUMS disrupted the circadian rhythm of Per2, Bmal1, Clock, and Per1, but not Cry2. Furthermore, immunostaining of Per2 also showed a loss of circadian rhythm in CUMS group (*p* = 0.133) (Fig. S7). Therefore, for the subsequent study, Per2 was selected for detailed investigation.

**Fig. 5:**
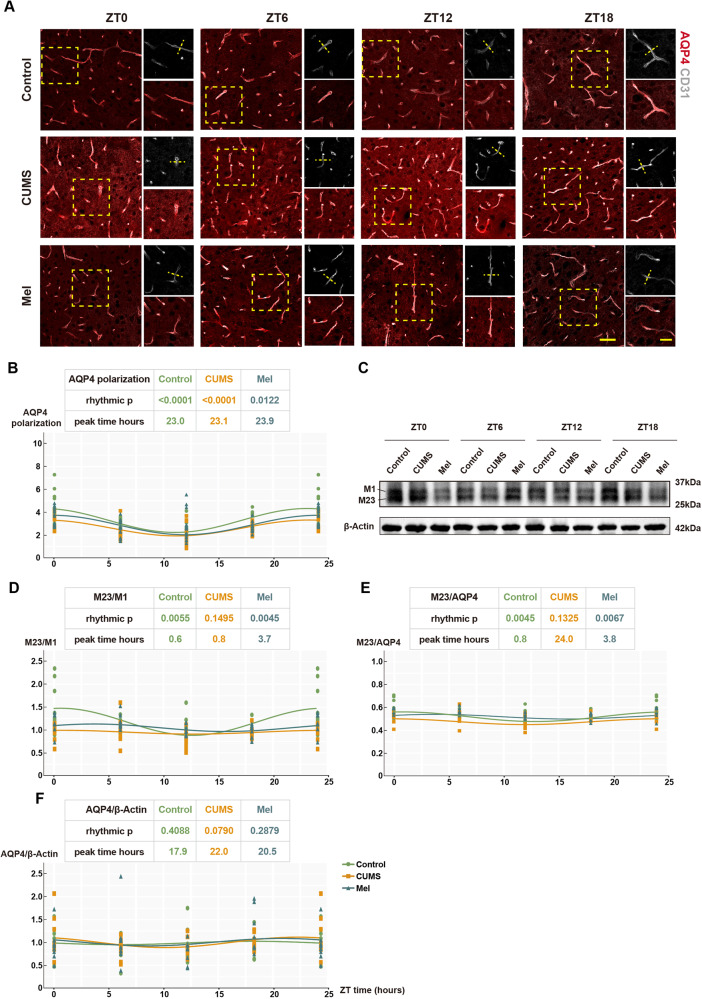
Melatonin treatment maintains/restores the AQP4 polarization in CUMS mice. **A** Representative image of AQP4 immunostaining at ZT0, ZT6, ZT12, and ZT18, respectively. Scale bar, 50 μm and 25 μm. **B** Quantitation of AQP4 polarization to (**A**). AQP4 polarization = vessel AQP4 (peak of the vertical line)/global AQP4. Each group *n* = 5. **C** Representative western blots of AQP4 M1 and M23 subtypes at ZT0, ZT6, ZT12, and ZT18, respectively. **D**–**F** Quantitation of AQP4 polarization to (**C**). Each group, *n* = 8.

Interestingly, the polarization of AQP4 also exhibits a rhythmicity, as recapitulated in the present study. The fluorescence immunohistochemistry results revealed that there was no evidence of disordered circadian rhythm in the polarization of AQP4 among the three groups (rhythmic *p*-value 0.05) (Fig. 5A, B). Previous studies have suggested that the expression of the M23 subtype of AQP4 is more likely to occur at the endfeet, whereas the M1 subtype is mainly expressed at the soma [18]. Therefore, the ratio of M23/M1 is considered as an indicator of AQP4 polarization [12]. In order to further investigate AQP4 polarization, we used western blot to analyze AQP4 and its subtypes. Our findings demonstrated that the CUMS group had disrupted circadian rhythmicity of M23/M1 (rhythmic *p*-value > 0.05), compared to the control group. However, treatment with melatonin normalized AQP4 polarization to control levels. Additionally, the ratio of M23/total AQP4, which is another index of AQP4 polarization, also indicated that AQP4 polarization was disturbed in the CUMS group, but was rescued with melatonin treatment. However, although the polarization AQP4 was impaired significantly in the CUMS group, and could be rescued by melatonin treatment, the total amount of AQP4 did not differ among the three groups (Fig. 5C–F). In summary, the circadian rhythmicity of AQP4 polarization occurs in concert with circadian protein expression. Impairments in these indices in the CUMS group were rescued by melatonin treatment.

**Fig. 6:**
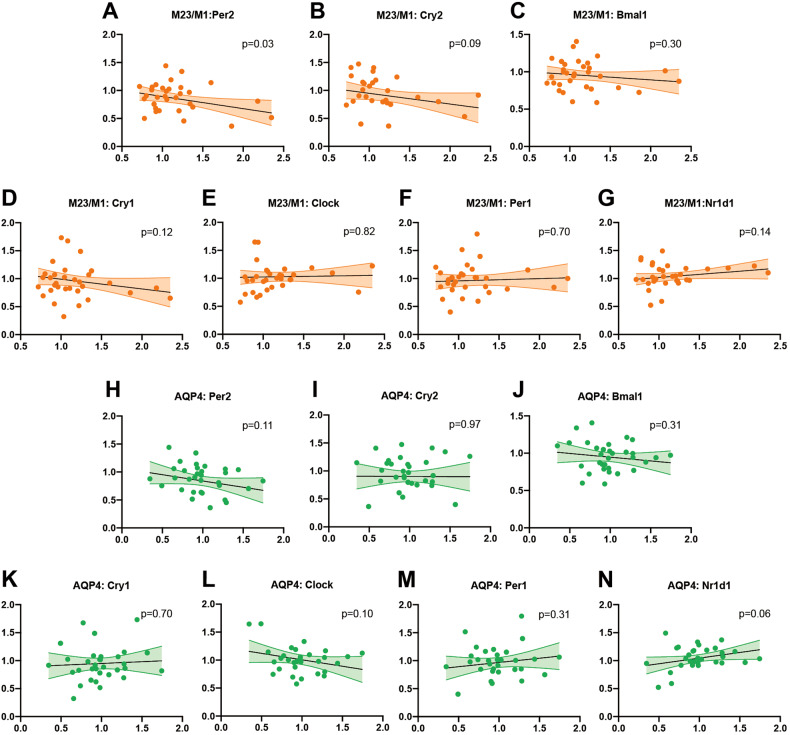
The expression of Per2 negatively correlated to the polarization of AQP4 but not the total amount of AQP4. **A**–**G** Correlation analysis of AQP4 polarization (M23/ M1) with brain expression of circadian proteins in the control group. **H**–**N** Correlation analysis of AQP4 amounts with circadian proteins in the control mice. *n* = 32. Colored zones are 95% confidence intervals.

We then analyzed the correlation between the expressions of circadian rhythm proteins and the polarization of AQP4 (M23/M1) in the control group across all time points. Of note, only the expression of Per2 and polarization of AQP4 (M23/M1) showed a significant negative correlation (*p* = 0.03), suggesting that the Per2 regulated the polarization of AQP4 negatively (Fig. 6A–G). Our analysis of correlations between the expressions of circadian rhythm proteins and the total amount of AQP4 in the control group did not indicate any significant relationships (Fig. 6H–N). These findings suggest that Per2 has the strongest correlation with AQP4 polarization.

### Per2 negatively regulates the polarization of AQP4 by Dtna

**Fig. 7:**
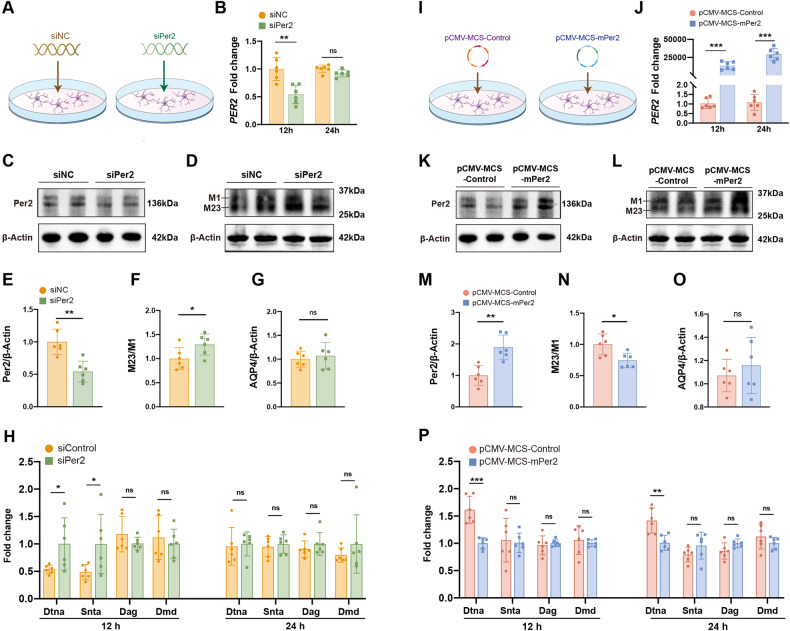
Per2 negatively regulates the polarization of AQP4 in primary cultured astrocyte. **A** Schematic diagram of siRNA interference in primary cultured astrocytes. **B** Quantitation of mRNA of PER2 by real-time PCR at 12 and 24 h after the interference. **C**, **D** Representative western blots of Per2 and AQP4 48 h after the interference. **E**–**G** Quantitation of (**C**, **D**). **H** Quantitation of the mRNA of DAC components by real-time PCR 12 and 24 h after the interference. **I** Schematic diagram of plasmid transfection in primary cultured astrocytes. **J** Quantifications of PER2 by real-time PCR 12 and 24 h after the transfection. **K**, **L** Representative western blots of Per2 and AQP4 proteins at 48 h after the transfection. **M**–**O** Quantitation of (**K**, **L**). AQP4 polarization = M23/M1. **P** Quantitation of the mRNA of DAC components by real-time PCR 12 and 24 h after the transfection. Each group *n* = 6. *<0.05, **<0.01, ***<0.001.

The localization of AQP4 to the vascular endfeet of astrocytes is dependent on DAC components (Dag1, Dmd, Dtna, Snta1), which have highly enriched gene expression in astrocytes according to a cell-specific transcriptome database [7]. The day/night changes in gene expression for components of this complex support rhythmic polarization of AQP4 [12]. We hypothesized that Per2 rhythmic regulates AQP4 polarization via DAC components. To test this, we used siPer2 interference in primary cultured astrocytes, which were interfered and harvested at 12 and 24 h, and assay for gene expression using RT-qPCR. Per2 mRNA and expression were effectively suppressed to 50% of control levels after 12 h of the interference, but did not differ significantly at the 24-hour timepoint (Fig. 7A, B). Western blot analysis of Per2 protein levels and AQP4 polarization at 48-hour after interference showed that suppressing Per2 expression induced a reduction of Per2 levels and an elevation of the M23/M1 ratio, suggesting that Per2 knockdown indeed leads to APQ4 polarization (Fig. 7C–G). Next, we measured gene expression of key components of the DAC including Dtna, Snta, Dag, Dmd. Interestingly, Dtna mRNA expression increased after silencing Per2, indicating that Dtna may be the DAC component whereby Per2 regulates the localized AQP4 expression (Fig. 7H). In another experiment, we upregulated the expression of Per2 in primary cultured astrocytes via transfection with a Per2-overexpression plasmid. RT-qPCR results showed that Per2 mRNA levels of a dramatical increase at 12 and 24 h after the transfection, whereas the Per2 protein level was increased at 48 h after the overexpression. In contrast with the results of Per2 silencing, the M23/M1 ratio was reduced, while the total amount of AQP4 remains unchanged in the overexpression cells. Among the DAC components, only Dtna had a reduced expression when Per2 was overexpressed (Fig. 7I–P). Taken together, our results further confirm that Per2 negatively regulates the polarization of AQP4.

### Melatonin protects CUMS mice from depression-like behaviors via AQP4

**Fig. 8:**
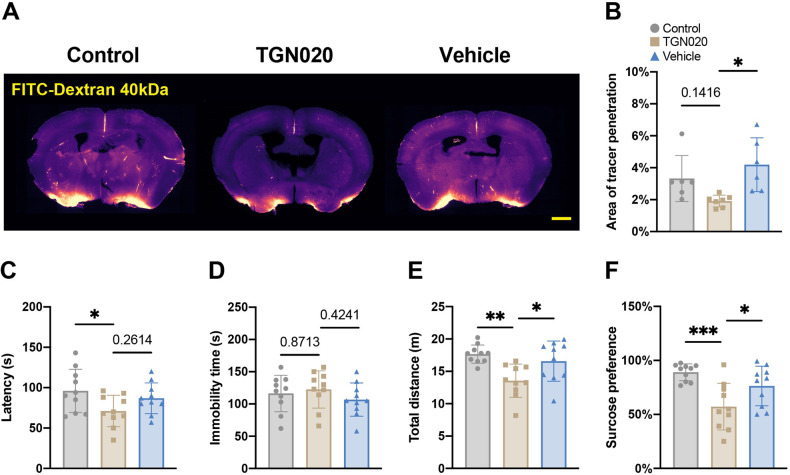
Blocking AQP4 with TGN020 impaired the function of the glymphatic system and resulted in depression-like behaviors. **A**, **B** Measurement of glymphatic function. Control, *n* = 6; TGN020, *n* = 7; Vehicle, *n* = 6. * <0.05. **C**, **D** The latency to the first immobility and the duration of total immobility in the tail suspension test (TST). **E** The total travel distance during the open field test (OFT). **F** The percentage of sucrose consumption during the sucrose preference test (SPT). *n* = 10 for each group. * <0.05, ** <0.01, *** <0.001.

To determine if melatonin protected CUMS mice from depression-like behaviors via AQP4, we pharmacologically blocked AQP4 with TGN020. Our findings suggested that inhibiting AQP4 impaired the glymphatic system and induced depression-like behaviors. Compared to control groups, blocking AQP4 resulted in a shorter latency to immobility and a longer duration of immobility in the TST, reduced travel distance in the OFT, and decreased sucrose consumption in the SPT (Fig. 8A-F).

## Discussion

We report in this study that protective effects of melatonin on depressive symptoms and cognitive impairment in CUMS depression model mice were mediated by alterations in glymphatic function and sleep structure. The CUMS mice showed disrupted circadian rhythmic expression of Per2, Bmal1, Clock, and Per1. Of note, Per2 expression had a negative correlation to AQP4 polarization. The main novelty of this study lies in our findings that melatonin treatment restores the effects of CUMS on glymphatic-system-function regulation by modulating the expression of the circadian rhythm protein Per2. Specifically, Per2 negatively regulated expression of the AQP4 subunit Dtna, which was apparently the driver for abnormal astrocytic AQP4 polarization in the CUMS model mice.

Depression is often associated with cognitive impairment, which is closely related to glymphatic dysfunction [13, 19]. The glymphatic system is a highly efficient metabolite clearance pathway, with well-described effects on the clearance of Aβ from brain [20]. This and previous studies have demonstrated that impaired glymphatic function in depression models arises in conjunction with decreased astrocyte numbers and loss of AQP4 polarization [13]. We augment earlier results by showing that melatonin treatment maintains the number of astrocytes and the polarization of AQP4 in CUMS mice, therefore restoring the fundamental structure and function of the glymphatic system, including influx and efflux functions. There is a recent report that glymphatic function in mice exhibits a daily rhythm, with a peak during their mid-day somnolent period [12]. The circadian rhythm of the glymphatic system is regulated in part by AQP4 perivascular vascular polarization in cortical astrocytes, which is supported by rhythmic gene expression of DAC protein. Present findings provide new insight into the mechanism whereby melatonin can improve depressive symptoms. We propose here that melatonin may restore more normal sleep architecture, thus maintain the circadian rhythmic activity of the glymphatic system, and thereby improves behavioral and cognitive measures.

Our study found that melatonin administration facilitated the clearance of Aβ40, but not Aβ42, indicating that these two forms of Aβ may cleared from the brain through distinct mechanisms. We suggested that the glymphatic system was more efficient in clearing soluble materials than insoluble ones, and that the balance between the glymphatic system and the catabolic pathway might contribute differently to the clearance of Aβ40 and Aβ42. It is important to note that Aβ42 is more prone to aggregation and considered more neurotoxic than Aβ40, which may explain its slower clearance. Previous research has also shown that the major catabolic pathway for Aβ42 in the brain involves neutral endopeptidase (NEP), a process similar to neprilysin, and that inhibiting NEP results in pathological deposition of Aβ42 in the brain [21]. Therefore, it is likely that the major clearance pathway for Aβ40 from the brain might through the CSF circulation, while the clearance of Aβ42 may rely more on catabolic pathways. Further research is necessary to fully understand the underlying mechanisms behind these observations of differential clearance of amyloid peptides.

To investigate this mechanism, we analyzed the effects of melatonin on the sleep structure of CUMS mice. Their sleep quality was affected mainly during the rest state, rather than in the nocturnal active state. The decrease in sleep time in CUMS mode mice was mainly due to decreased duration of individual sleep bouts, without any change in the number of sleep bouts. Melatonin treatment improved total sleep time and the amount of NREM sleep. During the rest state, CUMS mice showed increased power in the alpha and beta EEG bands, but only the increased beta power (12.5 and 30 Hz) was reversed by melatonin treatment. Insofar as beta power is linked to integration of sensory input and learning of new motor skills [22, 23], results of our study give valuable insights into how melatonin affects sleep structure in CUMS mice.

A previous study discovered that chronic mild stress led to changes in REMs before any other measured sleep characteristic [24]. In our study, we found that CUMS also led to a reduction in NREM duration, but it did not affect REM sleep percentage. The proportion of REM sleep during total sleep was approximately 15%, which aligns with Nollet’s findings. However, in a previous study, it was found that social defeat stress had a greater impact on reducing the duration of NREM sleep in C57 mice than REM sleep [25]. Another study showed that rats that experienced social conflict, both winners and losers, had increased slow wave activity during NREM sleep after the conflict but reduced REM sleep in the first few hours following the conflict [26]. Alternatively, we found that CUMS could potentially reduce the latency of REM while melatonin can alleviate the reduction in our experiments. These results suggest that the mechanisms underlying REM changes after stress are complex and require further investigation.

Sleep quality is highly dependent on circadian expression of certain proteins [27]. The transcription-translation negative feedback loop mechanism (TTFL) explains how circadian rhythms are maintained in animal cells [28]. Mammalian TTFL involves transcriptional activation of Clock/Bmal1 hetero-dimers, which drives the daytime expression of *Period* (*Per*) and *Cryptochrome* (*Cry*) genes via E-box regulatory sequences. Following dimerization and transport to the nucleus, Per-Cry complexes repress Clock-Bmal1 activity during the circadian night, until progressive degradation of Per-Cry allows the onset of a new cycle. The astrocytic TTFL alone can drive molecular oscillations in the suprachiasmatic nucleus and circadian behavior in mice [29].

In our study, CUMS caused a shift in the peak time of circadian protein expression. The melatonin treatment partially normalized the peak time of protein expression. Per2 and Bmal1 were rhythmic in antiphase (peak time = 12.8 h and 7.5 h respectively in our results). The expression of Per2 is statistically negatively correlated with M23/M1. Bmal1, as the antiphase protein of Per2, should be positively correlated with M23/M1. However, we did not observe this correlation, possibly because the antiphase of Bmal-Per2 did not have an exact 12-hour cycle.

The rhythmic expression of Per2, Bmal1, Clock, and Per1 was disrupted in CUMS mice. All these proteins were normalized by melatonin treatment, but we emphasize that only Per2 expression correlated with AQP4 polarization. Interestingly, the expression of Per2 correlated to the polarization of AQP4, which facilitates the functioning of the glymphatic system. Indeed, AQP4 shows a circadian polarization, with a zenith in polarization during the resting period, and depolarization during the active, waking state (Figs. 5, 6A).

We discovered in this study that the circadian gene Per2 diminishes the expression of Dtna, a component of the DAC known to regulate the polarization of AQP4 [30]. As such, it is intriguing to consider how Per2 influences AQP4 polarization. Circadian proteins play a critical role in controlling the rhythmic expression of circadian proteins. For instance, the Clock and Bmal1 proteins heterodimerize and bind to specific DNA sequences in the promoter regions of their target genes, which in turn activates their transcription. The transcriptional activity of these positive regulators (Clock and Bmal1) is then inhibited by their interaction with PER and CRY proteins, forming a negative feedback loop. Given these interactions, we can plausibly hypothesize that Per2 negatively regulates Dtna expression, which subsequently affects the M23/M1 ratio. However, the precise role of Per2 in establishing this ratio remains unclear. Among the possible explanations, Per2 might downregulate Dtna, thereby disrupting the localization of M23-AQP4 at the astrocytic endfeet. Alternately, the M23 isoform might be expressed independently of Dtna. Indeed, a previous study found that the expressions of M1 and M23 isoforms do not align with the levels of their respective mRNAs [31], which may suggest the existence of posttranscriptional regulation. The rhythmic modification of these isoforms might also be associated with the rhythmic expression of Per2, which is a matter for further investigations.

Stress is known to induce astrogliosis in several neurological diseases [32]. Nevertheless, our results combined with previous studies demonstrated that astrogliosis did not occur after CUMS [33, 34]. Histological research in depression patients also indicated that the number of astrocytes was decreased and morphological atrophy occurred in the prefrontal cortex, amygdala, and hippocampus [35, 36]. The underlying mechanisms may stand as follows. First, chronic social stress leads to the abnormal activation of the hypothalamic-pituitary-adrenal axis, which is reported to exert negative influences on astrocytic metabolism and lead to more pronounced atrophy [37]. Second, programmed cell death happens in astrocytes after CUMS [38]. Third, the death and atrophy of astrocytes consequently lead to a decrease of neurotrophins such as brain-derived neurotrophic factors [39]. Furthermore, the impaired function of the glymphatic system due to CUMS may exacerbate these effects, forming a vicious cycle. Melatonin is widely used in clinics for its triple effects, namely sleep-wake rhythm restoration, anti-inflammation, and neurotrophy [40]. More importantly, melatonin may exert its protective function on glymphatic system via alleviating sleep-wake rhythm disruption after CUMS as illustrated by our results.

In summary, we confirm herein that the circadian rhythm of AQP4 polarization is disrupted in CUMS mice, resulting in glymphatic system dysfunction. We link this to the perturbation of rhythmic Per2 expression, abnormal sleep architecture, and AQP4 polarization rhythms in CUMS mice. Melatonin treatment restored the AQP4 polarization and ultimately attenuated the CUMS-induced sleep disorder and associated behavioral outcomes and impairment in spatial memory.

## Methods

### Animals

The animal experiments were approved by the Experimental Animal Ethical Committee of Tongji Hospital affiliated to Huazhong University of Science and Technology and performed according to the ARRIVE guidelines. The numbers of animals for each group were chosen in accordance with similar previously published experiments. Adult male C57BL/6 mice (age 10-12 weeks) were fed in the Experimental Animal Center of Tongji Hospital. Mice were housed under standard specified pathogen-free (SPF) housing conditions with a 12 h light/dark cycle; the lights were turned on at ZT0 and turned off at ZT12. Mice had *ad libitum* access to food and water, except for specific periods during the modeling and behavioral testing. Before the experiments, mice were allowed to acclimate to the new surroundings for at least one week. After acclimation, mice were randomly divided into control, CUMS, and CUMS + melatonin treatment groups. Animals from different cages in the same experimental group were selected to ensure randomization. To exclude the effect of melatonin on baseline activity of mice, we measured the daily activity and compared the circadian rhythm after wild-type mice treated with melatonin. Our result showed that melatonin did not change the circadian rhythm and glymphatic system function in wild-type mice (Fig. S2A–D).

### CUMS modeling, melatonin, and TGN020 administration

The CUMS paradigm was administrated as described previously [13]. In brief, mice were exposed to different stressful stimuli in random sequences every day for two months. The stressors consisted of water deprivation for 12 h, food deprivation for 24 h, forced swimming in ice water for three min, cage tilting at 45° for 24 h, provision of damp bedding for 24 h, and tail clipping for five min.

Melatonin treatment was delivered from the fourth week of the CUMS paradigm. Melatonin (M5250, Sigma, German) was injected intraperitoneally at a dose of 10 µg per gram body weight every day at ZT0 for four weeks. Melatonin was dissolved in ethanol and then formulated in normal saline to a final concentration of 1.25 mg/ml.

For a specific illustration of AQP4 regulation by melatonin treatment, TGN020 (MCE, HY-W008574, China) was applied for pharmacological inhibition of AQP4 polarity according to a previous report [20]. In brief, the mice were divided randomly into three groups: control, CUMS+melatonin+TGN020, and CUMS+melatonin+vehicle. After four weeks of CUMS paradigm, the CUMS+melatonin+TGN020 group received an intraperitoneal injection of TGN020 (250 mg/kg in 20 ml/kg body weight) dissolved in 20% SBE-β-CD (MCE, HY-17031, China), while the CUMS+melatonin+vehicle group received only the vehicle (20% SBE-β-CD, 20 ml/kg) at the same dose.

### Tail suspension test (TST)

The TST was performed in a quiet laboratory as an index of behavioral despair, as previously described [13]. In brief, the tail of the mouse was taped on a crossbar elevated at 15 cm for five min. The time from the start of the test to the time when mice ceased struggling was recorded as the latency. The total time mice spent without any struggle during the test was recorded as the total immobility time.

### Open field test (OFT)

The OFT was performed to examine the anxiety state of the mice, as previously described [13]. In brief, the mouse was gently placed in the open field and allowed to explore for five min. After each exploration, the field was thoroughly cleaned with ethanol to eliminate olfactory cues left by the previous test animal. The exploration tracks were recorded with a video camera (Hikvision Digital Technology Co. Ltd., China) and analyzed by Any-Maze behavioral tracking system (Stoelting Co. Ltd., USA).

### Sucrose preference test (SPT)

The SPT was applied in a quiet environment to assess anhedonia, as described previously [13]. In brief, mice were acclimated for 48 h to two water bottles containing 1% sucrose solution in the cage. After the 24-hour deprivation of water, each cage was fitted with bottles containing water and 1% sucrose solution, respectively. The consumption of plain water and sucrose solution during the following 12 h test with ad libitum access to both bottles were recorded and calculated as the index for the sucrose preference.

$$\begin{array}{l}{\rm{Sucrose}}\,{\rm{preference}}( \% )\\={\rm{sucrose}}\; {\rm{solution}}\; {\rm{consumption}}({\rm{g}})/{\rm{total}}\; {\rm{fluid}}\; {\rm{consumption}}({\rm{g}})\times 100 \%\end{array}$$

### Morris water maze (MWM)

The MWM test was performed as previously described [41]. In brief, mice were trained for five days for spatial memory acquisition, and subjected to the probe test on day six. During the acquisition period, mice were gently placed into the opaque water-filled maze and allowed to explore freely for one minute. If the mice discovered the hidden platform, they were allowed to stay on the platform for an additional 30 s to afford consolidation of spatial memory cues. Otherwise, mice were guided onto the platform and allowed to stay there for 30 s before removal to their home cage. During the probe test, the platform was removed and the mice were initially placed in the quadrant opposite the quadrant where the platform had stood. The swimming tracks were recorded with a video camera (Hikvision Digital Technology Co. Ltd., China) and analyzed by Any-Maze behavioral tracking system (Stoelting Co. Ltd., USA).

### Intracisternal tracer injection and fluorescence imaging

The CSF tracer FITC-Dextran 40 kDa Lysine Fixable (Invitrogen, USA) was dissolved in the artificial CSF at a concentration of 1% (w/v). For the anesthesia state, mice were anesthetized with pentobarbital (1%, intraperitoneally (i.p.)). After the disappearance of the tail-pinch pain reflex, the scalp was disinfected and the cisterna magna was surgically exposed as described previously [41]. A 30G-needle was gently inserted into the cisterna magna, and the prepared CSF tracer was infused at a rate of 1 µl/min for ten minutes by a syringe pump (Harvard Apparatus, USA). Thirty minutes after the infusion, mice were transcardially perfused first with normal saline, and then with pre-cold 4% paraformaldehyde solution. For the awake state, the 30 G needle was pre-implanted. Here, the mice were anesthetized with pentobarbital (1%, i.p) and the needle was inserted into the cisterna magna and fixed in place with super glue and dental cement on the skull. On the next day, when the mice were totally recovered from anesthesia, the prepared CSF tracer was infused via the implanted needle at a rate of 1 µl/min for then minutes. Half an hour later, the mice were transcardially perfused as described above. The brain samples were post-fixed and dehydrated in 30% (w/v) sucrose solution twice time.

For brain-wide fluorescent tracer imaging, a laser zoom-stereo microscope (Nikon, SMZ18, Japan) was applied to capture images. For fluorescent tracer imaging in brain slices, brain samples were sliced to 100 μm thickness with a cryotome (Leica, CM1950, German) and scanned with an upright slice-scanning microscope (Nikon, ECLIPSE Ni-E, Japan). The resultant fluorescent images were analyzed using Fiji software by an investigator who was blinded to the sample identity.

### Stereotactic injection of exogenous human Aβ mixture and ELISA

These glymphatic efflux procedures were performed as previously described [41]. In brief, mice were anesthetized with 1% pentobarbital (i.p.) and placed in a stereotaxic frame. A mixture of human Aβ40 (0.0433 mg/L) and Aβ42 (0.0451 mg/L) peptides (Chinapeptides, China) was injected into the left prefrontal cortex (2 mm AP, 1 mm lateral, and 0.75 mm deep relative to the bregma). After an infusion at a rate of 0.05 µL/min for ten minutes, the needle was left in place for ten minutes prior to removal. Mice were sacrificed one hour after the end of the injection, and the left cerebral hemisphere was collected. For the Aβ analysis, tissues were first homogenized in 1 ml/g PBS and then assayed using human ELISA kits for Aβ40 (CSB-E08299h, Cusabio, China) and Aβ42 (CSB-E10684h, Cusabio, China).

### Immunofluorescence staining

Mice were anesthetized with 1% pentobarbital (i.p.) and sacrificed by transcardial perfusion while deeply anesthetized, as described above. The post-fixed brains were sliced to 30 μm thickness using a cryotome (Leica, CM1950, German). The collected slices were first incubated for 15 min in immunostaining permeabilization buffer containing Triton X-100 (P0096, Beyotime, China), followed by 15 min in immunostaining blocking buffer (P0260, Beyotime, China), and finally in buffer containing primary antibodies, namely mouse anti-GFAP (1:200; 3670, CST, USA), rabbit anti-AQP4 (1:50; 16473-1-AP, Proteintech, China), rabbit anti-AQP4 (1:200; A13168, ABclonal, China), and rat anti-CD31 (1:200; 550274, BD Biosciences, USA) overnight at 4 °C. After rinsing three times in PBS, slices were incubated with corresponding secondary antibodies, including Alexa Fluor 647-conjugated donkey anti-mouse (1:400; Abcam, USA), Cy3-conjugated goat anti-rabbit (1:400; Jackson Immunoresearch, USA), and Alexa Fluor 488-conjugated donkey anti-rat (1:400; Jackson Immunoresearch, USA) for one hour at room temperature. After dehydration, slide mounting, and clearing the immunofluorescent images were captured by laser scanning confocal microscopy (Olympus, FV1000, Japan) and analyzed using Fiji software by a blinded investigator.

### AQP4 polarization analysis

In general, three methods were used repeatedly during the experiments to measure the polarization of AQP4, which is important to the final conclusion. For immunofluorescence imaging analysis, we defined the extent of AQP4 polarization according to two patterns. One pattern was calculated from DAPI, GFAP and AQP4 immunostaining as previously described [41]. In brief, a donut-shaped area was drawn within 5 pixels from a capillary, which encompassed the void surrounded by zones enriched in DAPI and GFAP expression. The ratio of AQP4 immunofluorescence within the void to the global AQP4 signal indicated AQP4 polarization: *AQP4 polarization* *=* *donut-shaped area AQP4 / global AQP4*.

As an alternative method, we calculated polarization from the immunofluorescence signals of AQP4 and the vascular marker CD31. As described previously, we randomly selected a line perpendicular to a vessel, and recorded the fluorescent intensity along the line [42]: *AQP4 polarization* *=* *vessel AQP4 (peak of the vertical line) / global AQP4*.

In addition, according to the western blot analysis, we defined AQP4 polarization as the ratio of M23 to M1 subtype levels, also as previously described [12]: *AQP4 polarization* = *M23 / M1*, or *AQP4 polarization* = *M23 / (M1* + *M23)*.

### EEG and EMG acquisition and analysis

Mice were anesthetized with pentobarbital (1%, i.p.), and upon loss of tail-pinch reflect, were placed in a stereotaxic frame for head fixation. The skull was exposed as described above, and a headmount (8201, Pinnacle, USA) was fixed on the skull surface with super glue and dental cement. We then inserted EEG electrodes into the skull at these fixation points, and implanted EMG electrodes into the dorsal cervical muscles. After recovering from the anesthesia, mice were housed singly in a Plexiglas barrel measuring 40 cm in diameter, as described previously [43]. Mice were allowed to acclimate to the novel surroundings and the headmount for one week. The EEG and EMG signals were amplified and captured by Sirenia Acquisition software (Pinnacle, USA) from ZT0 to ZT24, and were analyzed by a blinded investigator using Sirenia Sleep Pro software (Pinnacle, USA) as previously described [44, 45].

### Western blot

Mice were sacrificed while deeply anesthetized with 1% pentobarbital (i.p.). Brain samples were harvested and homogenized with RIPA lysis buffer (G2002, Servicebio, China) containing a proteinase inhibitor cocktail (G2006, Servicebio, China), and then centrifuged at 12,000 rpm for 15 min at 4 °C. Loading buffer (G2013-100ML, Servicebio, China) was added to the collected supernatants, followed by mixing then then heating at 90 °C for ten minutes. After electrophoresis in SDS-PAGE hydrogel, proteins were transferred to nitrocellulose filter membranes of 0.45 µm pore size, which were then incubated overnight at 4 °C in the primary antibodies. There consisted of i.e. rabbit anti-Clock (1:1000; A7265, ABclonal, China), rabbit anti-Bmal1 (1:1000; 14268-1-AP, Proteintech, China), rabbit anti-Cry1 (1:1000; 13474-1-AP, Proteintech, China), rabbit anti-Cry2 (1:1000; 13997-1-AP, Proteintech, China), rabbit anti-Per1 (1:1000; 13463-1-AP, Proteintech, China), rabbit anti-Per2 (1:1000; A13168, ABclonal, China), rabbit anti-Nr1d1 (1:1000; 14506-1-AP, Proteintech, China), rabbit anti-AQP4 (1:1000; 16473-1-AP, Proteintech, China), and rabbit anti-β-Actin (1:2000; 23660-1-AP, Proteintech, China). After washing three times in TBST, we added the secondary antibody, Goat anti-Rabbit HRP (1:2000, GB23303, Servicebio, China), and incubated the membranes for one hour at room temperature. The expression level of the specific proteins was captured by the Chemiluminescence Imaging Analysis System (Biolight, China). Acquired band images were analyzed by a blinded investigator using Fiji software.

### Circadian rhythm analysis

We conducted analysis of the circadian proteins and daily activity using the ‘circacompare’ R package, as described in a published paper [46]. The code is available on GitHub at https://github.com/RWParsons/circacompare/. To summarize, the circadian protein expression at each time point was fitted to a cosine curve. The parameters, such as peak time and amplitude, of these curves can be used to describe the characteristics of the circadian rhythm. A circadian rhythm was indicated by a rhythmic *p*-value below 0.05.

### Primary astrocytes culture

Primary astrocytes were isolated and cultured as previous described, with minor modifications [47]. In brief, cerebral cortical tissues were aseptically extracted from C57BL/6 J mouse pups (P1–P3) and the meninges were carefully removed. Tissues were minced finely and digested with 0.25% trypsin for 30 min at 37 °C. Isolated cells were cultured with Dulbecco’s Modified Eagle’s Medium and Ham’s F12 medium (DMEM/F12, Gibco, USA) plus 10% fetal bovine serum (FBS, Boster, Chia) in T75 flasks. The culture medium was exchanged after 24 h. At day three, the culture medium was replaced with fresh DMEM/high glucose (DMEM/F12, Gibco, USA) plus 10% FBS and changed every three days thereafter. Cultures were incubated at 37 °C and 5% CO_2_. Primary astrocytes were purified by shaking at 220 rpm overnight. After removal of the supernatant, 0.25% trypsin was applied for digestion, and the supernatant was collected for centrifugation. Purified astrocytes were plated in six-well plates at a density of 4 × 10^5^ cells/well.

### Small interfering RNAs and plasmids transfection

Small interfering RNAs (siRNAs) and plasmids were purchased from Vigene Bioscience Inc, China. For Per2 inhibition, astrocytes are transfected with Per2 siRNA (siPer2) or negative control siRNA (siNC) using the Lipofectamine 3000 kit (Invitrogen) according to the manufacturer’s protocol. For Per2 overexpression, Per2 expression plasmid (pCMV-MCS-Per2, 5’CGCAAATGGGCGGTAGGCGTG, 3’GAAATTTGTGATGCTATTGC) and control miRNA expression plasmid (pCMV-MCS-Control) were transfected using Lipofectamine 3000 kit. After transfection for 12 and 24 h, cells are collected for PCR assays. After transfection for 48 h, cells are collected for western blot analysis.

### Real-time PCR

Total RNA from astrocytes was extracted with Trizol (9108, Takara, Japan). cDNA was reverse transcribed from 1 μg of RNA using HiScript II Q RT SuperMix for qPCR (R222, Vazyme, China) according to the instructions provided by the manufacturer. Quantitative RT-PCR was performed using ChamQ Universal SYBR qPCR Master Mix (Q711, Vazyme, China) in the Real-time PCR system (CFX96, BioRad) by the 2ΔΔ^Ct^ method. The expression levels of target genes were normalized to β-actin.

### Statistical analysis

Blinded scoring of behavioral tests, EEG/EMG signals, and AQP4 polarization was performed by investigators that were unaware of the experimental groups. Data were analyzed using GraphPad Prism 8.0 (GraphPad Software Inc., La Jolla, CA, USA). The Shapiro-Wilk test was first applied to confirm the normality of the data. *F*-test was used to compare variances between different groups. When data followed a normal distribution, we adopted the Student’s *t*-test for the comparation of two groups, and one-way analysis of variance (ANOVA) with Tukey’s post-hoc test for comparisons between multiple groups. For Morris water maze test, Two-way Repeated Measures ANOVA to compare three groups. Pearson correlation coefficients were calculated for correlation analyses. The results were expressed as mean ± SD. *P*-values less than 0.05 were considered as statistically significant differences.

### Supplementary information

Supplemental Material
