# Supplementary material for: Melatonin alleviates depression-like behaviors and cognitive dysfunction in mice by regulating the circadian rhythm of AQP4 polarization
Source: Transl Psychiatry. 2023 Oct 6;13:310. doi: 10.1038/s41398-023-02614-z (PMC10558463; doi:10.1038/s41398-023-02614-z)
Supplement: Supplementary file 1 — Supplemental Material [file 41398_2023_2614_MOESM1_ESM.docx]

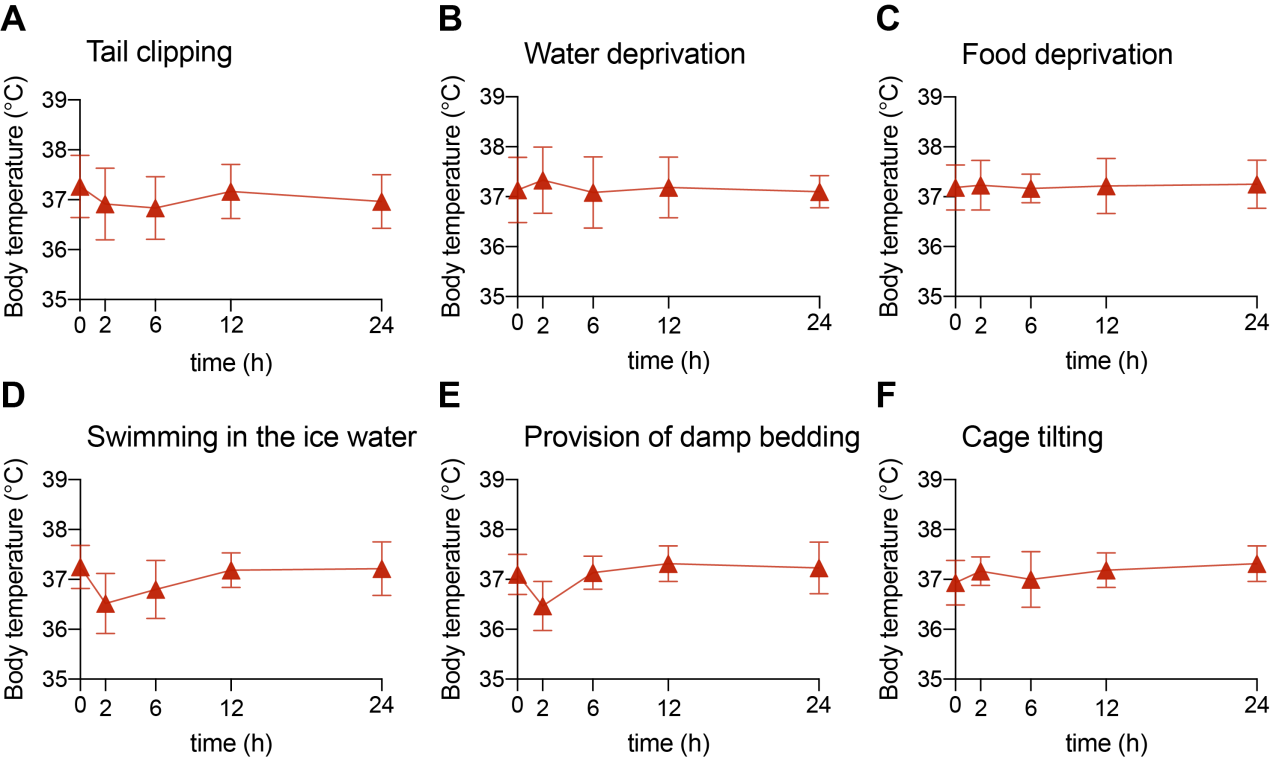


**Fig S1. The anal temperature after CUMS protocol.**

**A-F**. The anal temperature after tail clipping, water deprivation, food deprivation, swimming in ice water, providing damp bedding, and tilting of the cage. n=6.


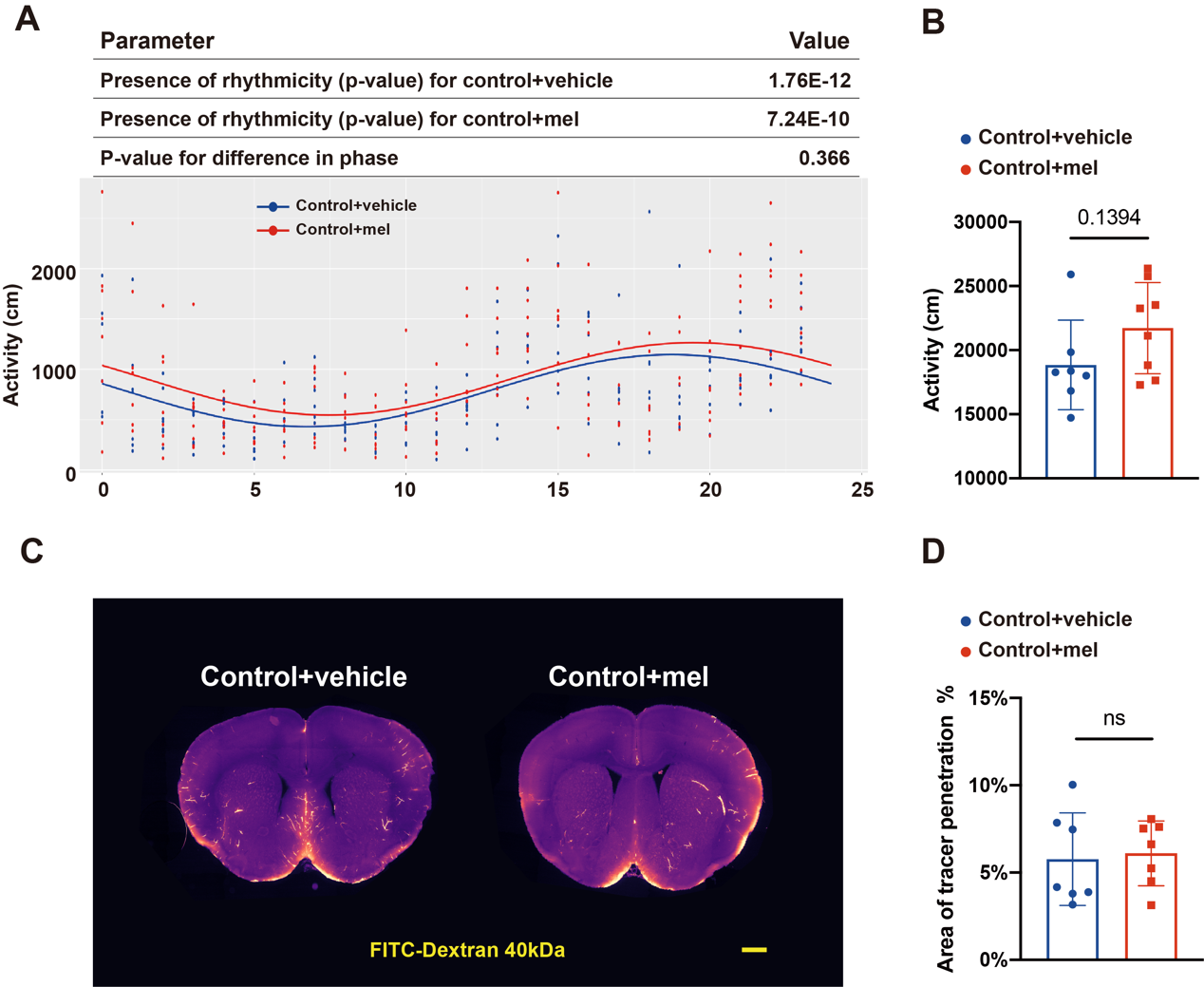


**Fig S2. The administration of melatonin alone did not change the circadian rhythm and the function of glymphatic system.**

**A.** Circadian rhythm was assessed using a rhythm monitor system. The mouse’s activity was measured every hour of the day, and a cosine curve was used to fit the data. Analysis using the circaCompare R package showed that both the control+vehicle and control+mel groups exhibited rhythmic activity (p<0.01 for both groups) and had no significant differences in phase (p-value for phase difference = 0.366). **B.** the level of activity was comparable between the two groups. n=7 for control+vehicle and n=8 for control+mel. **C-D.** Representative images of CSF tracers in the coronal slices of control+vehicle and control+mel groups. Scale bar, 2 mm. n=7.


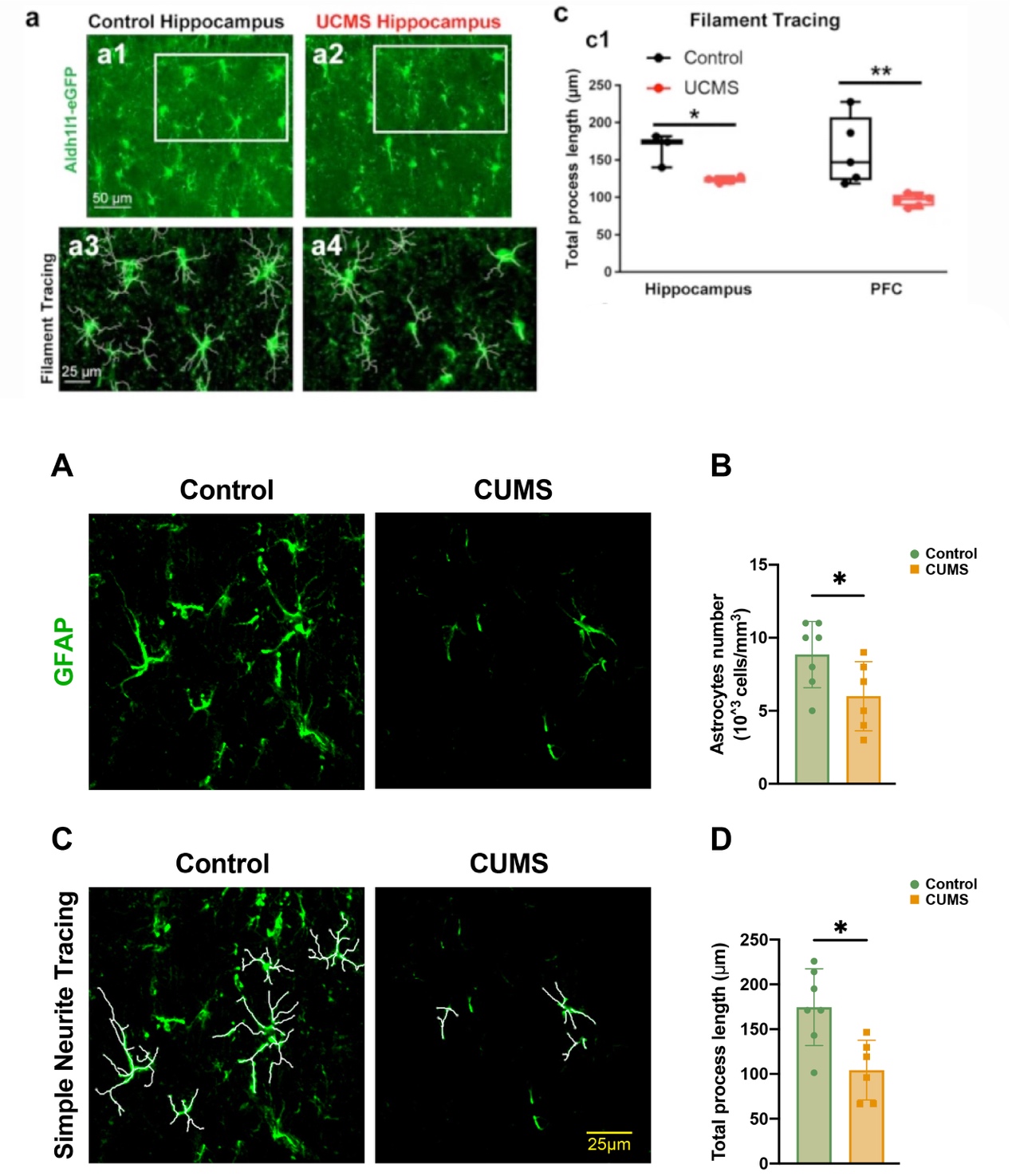


**Fig S3. The astrocyte processes were shortened in CUMS.**

**A-B.** The number of GFAP positive astrocytes in control and CUMS groups. Scale bar, 25 μm. n=7 for control and n=6 for CUMS.

**C-D.** The astrocyte processes were traced manually using the Simple Neurite Tracer plugin in Fiji. Scale bar, 25 μm. n=7 for control and n=6 for CUMS.


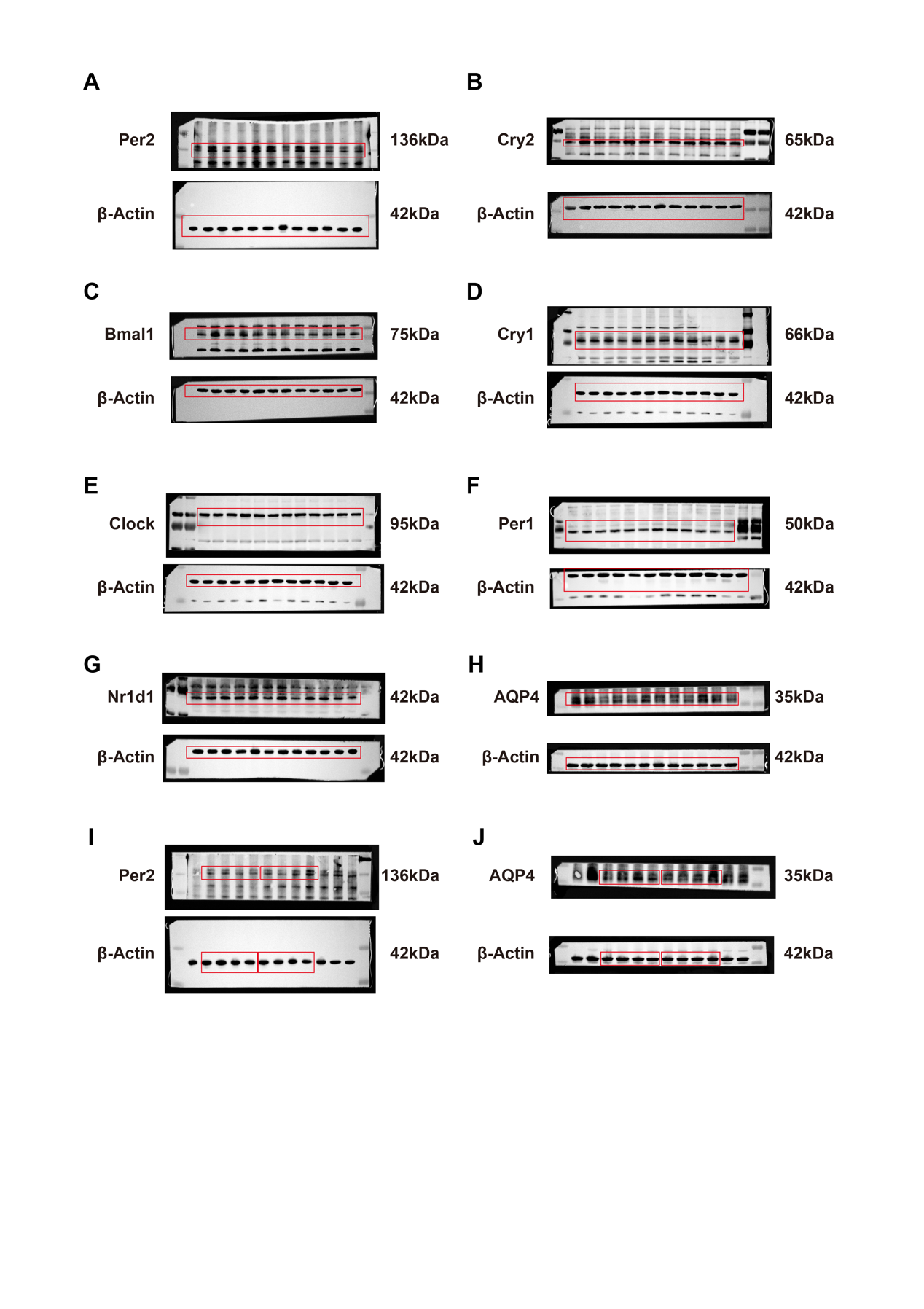


**Fig S4. Full-length membranes of western blot results in Figure 4 and Figure 5.**


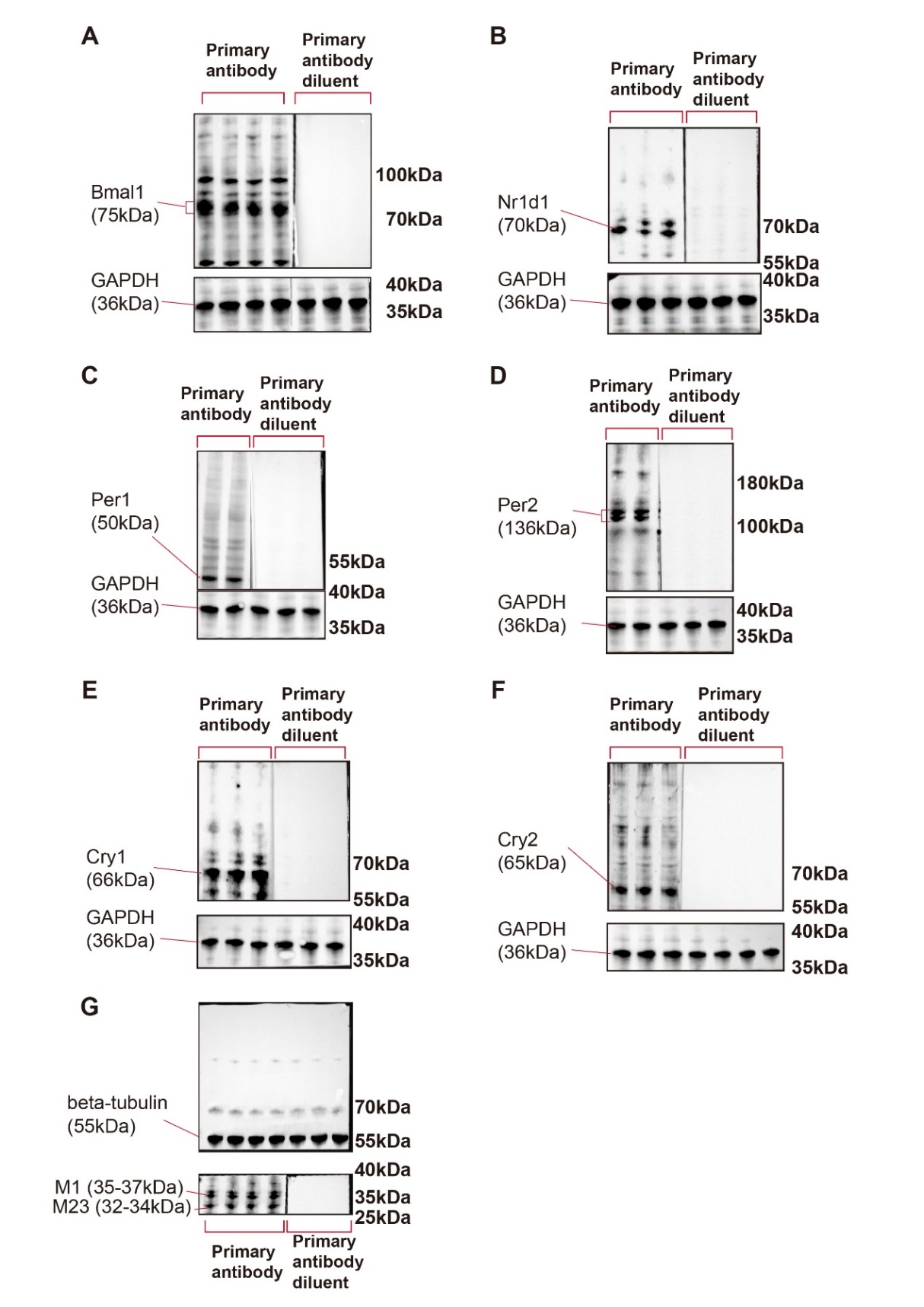


**Fig S5. Validations of antibodies by incubated with/ without primary antibodies.**

Western blot analysis was conducted on brain tissue samples from wild-type mice. The left panels of A-G showed the WB results obtained using specific primary antibodies for Bmal1, Nr1d1, Per1, Per2, Cry1, Cry2, and AQP4. The right panels of A-G correspond to the WB results obtained using the primary antibody diluent.


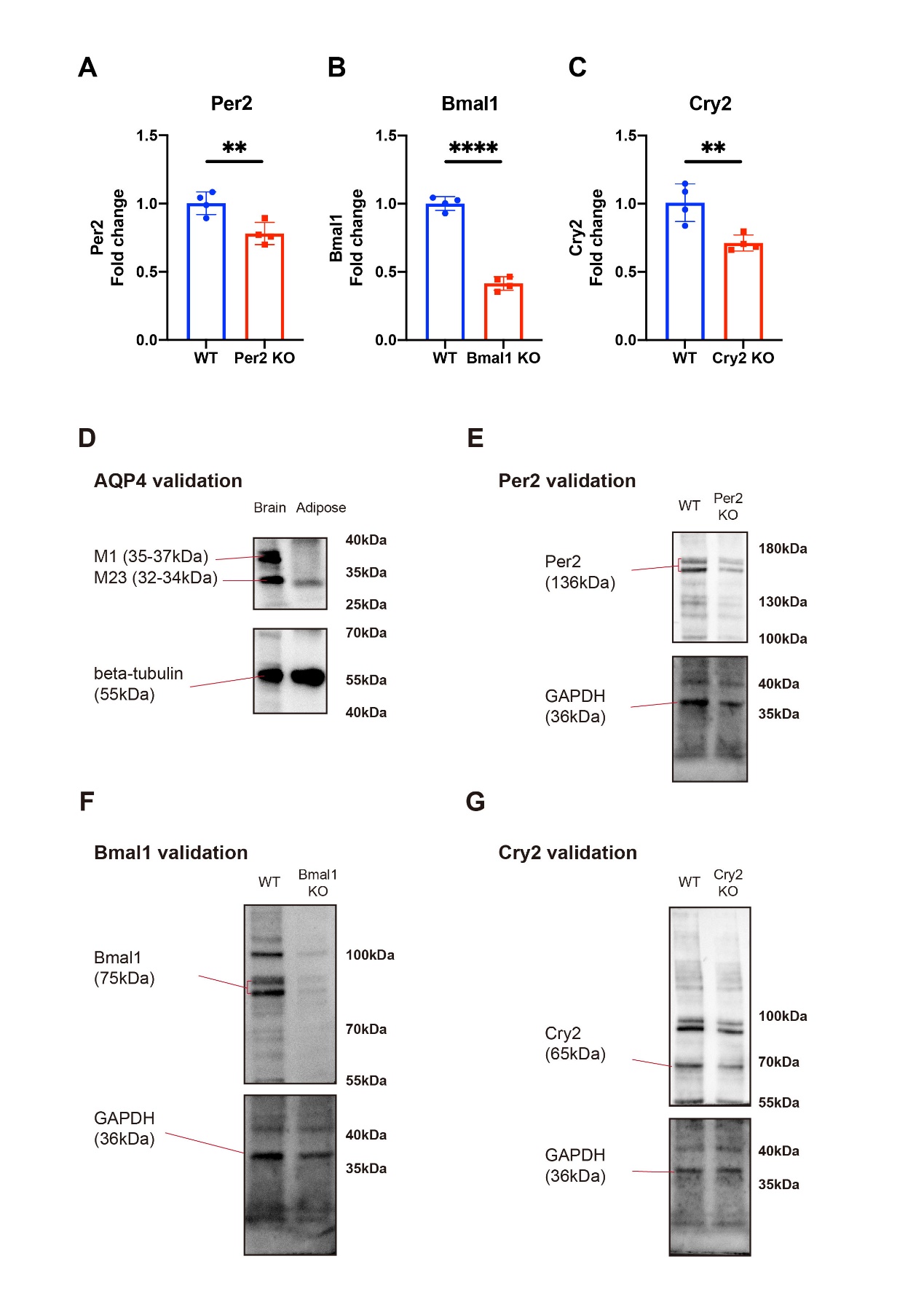


**Fig S6. Validations of antibodies by adipose tissue and knockout mice.**

**A.** Relative levels of Per2, Bmal1, and Cry2 in wild-type mice and knockout mice (Per2 knockout, Bmal1 knockout, and Cry2 knockout mice) brain measured by real-time PCR. n=4 for each group. ** <0.01, *** <0.001. **D.** Western blot result of AQP4 in the brain and adipose tissue of wild-type mice.

**E-G.** Western blot result of Per2, Bmal1, and Cry2 in wild-type mice and knockout mice.

**
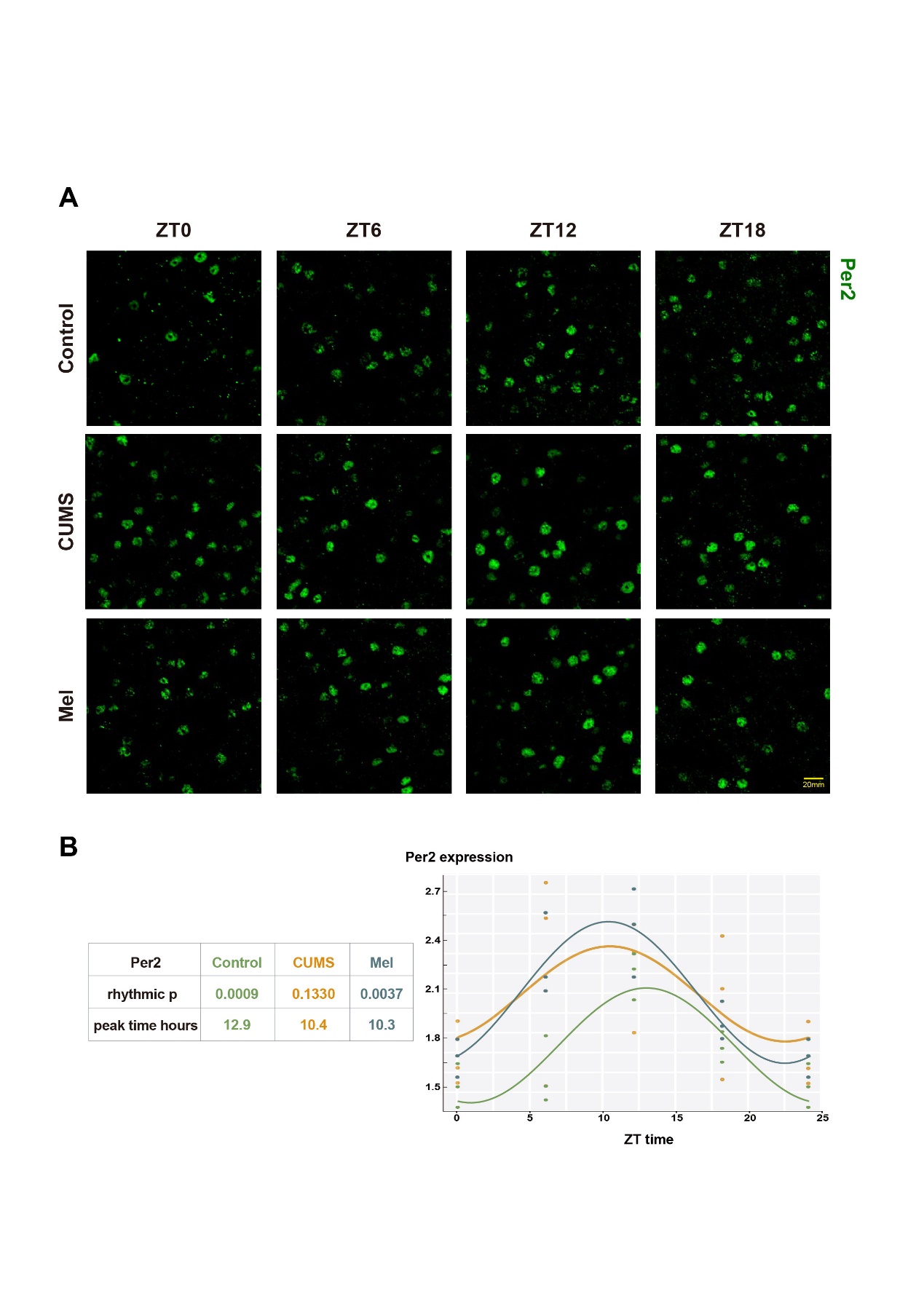
**

**Fig S7. Melatonin treatment rescues the disturbances of Per2 expression in CUMS mice.**

**A.** Representative images of Per2 immunostaining in prefrontal cortex in control, CUMS, and Mel group at ZT0, ZT6, ZT12, and ZT18, respectively. Scale bar, 20mm. **B**. Circadian rhythmicity of Per2 in **A**. Three images were averaged for calculation at each timepoint. n=3 for each group.
